# *De Novo* Assembly and Transcriptome Analysis of the Rubber Tree (*Hevea brasiliensis*) and SNP Markers Development for Rubber Biosynthesis Pathways

**DOI:** 10.1371/journal.pone.0102665

**Published:** 2014-07-21

**Authors:** Camila Campos Mantello, Claudio Benicio Cardoso-Silva, Carla Cristina da Silva, Livia Moura de Souza, Erivaldo José Scaloppi Junior, Paulo de Souza Gonçalves, Renato Vicentini, Anete Pereira de Souza

**Affiliations:** 1 Centro de Biologia Molecular e Engenharia Genética (CBMEG) - Universidade Estadual de Campinas (UNICAMP), Cidade Universitária Zeferino Vaz, Campinas, São Paulo, Brazil; 2 Agência Paulista de Tecnologia dos Agronegócios, Pólo Regional Noroeste Paulista, Votuporanga, São Paulo, Brazil; 3 Instituto Agronômico de Campinas (IAC), Campinas, São Paulo, Brazil; 4 Departamento de Biologia Vegetal, Instituto de Biologia, Universidade Estadual de Campinas (UNICAMP), Cidade Universitária Zeferino Vaz, Campinas, São Paulo, Brazil; RIKEN Biomass Engineering Program, Japan

## Abstract

*Hevea brasiliensis* (Willd. Ex Adr. Juss.) Muell.-Arg. is the primary source of natural rubber that is native to the Amazon rainforest. The singular properties of natural rubber make it superior to and competitive with synthetic rubber for use in several applications. Here, we performed RNA sequencing (RNA-seq) of *H. brasiliensis* bark on the Illumina GAIIx platform, which generated 179,326,804 raw reads on the Illumina GAIIx platform. A total of 50,384 contigs that were over 400 bp in size were obtained and subjected to further analyses. A similarity search against the non-redundant (nr) protein database returned 32,018 (63%) positive BLASTx hits. The transcriptome analysis was annotated using the clusters of orthologous groups (COG), gene ontology (GO), Kyoto Encyclopedia of Genes and Genomes (KEGG), and Pfam databases. A search for putative molecular marker was performed to identify simple sequence repeats (SSRs) and single nucleotide polymorphisms (SNPs). In total, 17,927 SSRs and 404,114 SNPs were detected. Finally, we selected sequences that were identified as belonging to the mevalonate (MVA) and 2-C-methyl-D-erythritol 4-phosphate (MEP) pathways, which are involved in rubber biosynthesis, to validate the SNP markers. A total of 78 SNPs were validated in 36 genotypes of *H. brasiliensis*. This new dataset represents a powerful information source for rubber tree bark genes and will be an important tool for the development of microsatellites and SNP markers for use in future genetic analyses such as genetic linkage mapping, quantitative trait loci identification, investigations of linkage disequilibrium and marker-assisted selection.

## Background

Natural rubber is one of the most important polymers that is produced by plants. Rubber is composed of 94% cis-1,4- polyisoprene and 6% proteins and fatty acids [1] and exhibits unique properties including flexibility, impermeability to liquids and abrasion resistance. These singular properties make natural rubber superior to synthetic rubber for use in various applications [2].

Natural rubber is used in more than 40,000 products, including over 400 medical devices, and is of great importance in the tire industry [2]. Approximately 2,500 plant species are known to synthesize natural rubber, but only a few plants, such as *Hevea brasiliensis* (rubber tree), *Parthenium argentatum (*guayule) and *Taraxacum koksaghyz* (Russian dandelion), can produce high-quality natural rubber with molecular weights of greater than 1 million Daltons [3]. Among these species, *H. brasiliensis* (Willd. ex Adr. de Juss.) Muell. –Arg., which is commonly referred to as the rubber tree, is the major source of natural rubber [2] and is planted on a large scale in fields encompassing approximately 11.33 million hectares [4].

*H. brasiliensis*, which is native to Amazon rainforests, is a diploid (2n = 36, n = 18), perennial, monoecious, cross-pollinated tree species [5], with an estimated haploid genome estimated of 2.15 Gb [6]. The genus *Hevea* belongs to the Euphorbiaceae family, which is comprised of 11 inter-crossable species [7].

Although the Amazon rainforest offers optimal conditions for growth and high rubber yields due to its warm and humid climate, this region also provides optimal conditions for South American leaf blight (SALB) disease, which is caused by the fungus *Microcyclus ulei* (P. Henn.) v. Arx. and was responsible for devastating plantations in northern Brazil in the 1930s. SALB remains a permanent threat to the rubber industry [8]. Because of this disease, rubber tree plantations have expanded throughout the world, in locations such as northeastern India, the highlands and coastal areas of Vietnam, southern China and the southern plateau of Brazil [9]. These areas are colder and drier than the Amazon rainforest and are not favorable for the growth of this fungus. However, they are associated with other types of stresses, such as low temperatures, strong winds and drought, that are limiting factors for rubber production [5]. Thus, rubber tree breeding programs have focused not only on genotypes that are resistant to SALB disease but also on those that are tolerant to the stress conditions found in these areas and are producers of high quality rubber.

Similar to many perennial trees, rubber tree breeding is time consuming and expensive. An average of 25 to 30 years of field experiments in large areas is generally required to obtain a new cultivar. Thus, molecular biological techniques could optimize field evaluations, thereby reducing the time and area that are required for these experiments.

Over the past two decades, there has been an exponential increase in data acquisition pertaining to the rubber tree, including with regard to genomic microsatellite markers [10], [11], expressed sequence tag-simples sequence reapeats (EST-SSRs) [12]–[14], linkage maps [15], [16] and gene expression profiles [17], [18]. More recently, a draft genome of the rubber tree was published [19]. High-throughput genomic techniques are associated with innovative bioinformatics tools that can be important to rubber tree breeding and facilitate the development of superior clones that are suited to different agroclimatic conditions [4].

With the reduction in the cost of next generation sequencing (NGS) technologies, RNA sequencing (RNA-seq) has become wide spread because it enables the high-resolution characterization of transcriptomes. This method provides many advantages, including a single-base resolution, enabling the detection of thousands of single nucleotide polymorphisms (SNPs) for further SNP marker development. These markers can be useful for the functional saturation of linkage maps and the identification of markers that are directly related to economic traits for marker assisted selection (MAS). In addition, RNA-seq can be employed to provide information about alternative splicing, to detect rare transcripts and to quantify different levels of expression of individual genes rather than total gene expression, in contrast with microarrays [20].

RNA-seq has become a valuable tool that has been used in the investiagation of many species, such as *Arabidopsis* [21], rice [22] and maize [23]. This technology has also been widely used in non-model species such as the rubber tree [24].

A search for *H. brasiliensis* in the National Center of Biotechnology Information (NCBI) revealed that approximately 40,000 EST sequences had been deposited (as of August 2013). Recently, a transcriptome profile for a mixture of leaves and latex was described [25] in addition to, a bark transcriptome and EST-SSRs markers have been developed [14]. Both of these studies used Illumina HiSeq 2000 technology. RNA-seq employing 454 pyrosequencing technology has also been applied to evaluate the apical meristem transcriptome to facilitate the development of EST-SSR markers and the construction of a genetic linkage map [13].

In the current study, a total of 166,731,798 high-quality reads from bark samples from the GT1 and PR255 clones were obtained through paired-end sequencing using Illumina GAIIx platform to generate a *de novo* assembly. The GT 1 clone, which is male-sterile, and PR 255 are good latex producers in São Paulo State and are parental to two mapping populations. These clones high yielding and cold and wind tolerant, which are important characteristics for rubber tree breeding. The obtained transcripts were submitted for functional annotations, through which it was possible to identify new genes in the *H. brasiliensis* database. The transcripts were also submitted for putative SSR and SNP discovery. A total of 78 SNP markers were validated in the mevalonate (MVA) and 2-C-methyl-D-erythritol 4-phosphate (MEP) pathways, which are two important pathways that are involved in rubber biosynthesis.

## Materials and Methods

### Ethics statement

We confirm that no specific permits were required for the present study. This work was a collaborative research project that was developed by researchers from the University of Campinas (UNICAMP) and the Agronomic Institute of Campinas (IAC). In addition, we confirm that the field study did not involve endangered or protected species.

### Plant materials, and DNA and RNA extractions

Bark samples from the GT1 and PR255 clones were collected at the Agência Paulista de Tecnologia dos Agronegócios/SAA, Votuporanga, São Paulo, Brazil. The selected clones were 18 years old and were tapped once every 4 days. The bark samples were frozen on dry ice and stored at −80°C until use. Total RNA was extracted according to Changet et al. [26]. RNA quality and integrity were evaluated using an Agilent 2100 Bioanalyzer (Agilent Technologies, Palo Alto, CA).

To validate the SNP markers, genomic DNA from 36 genotypes of *H. brasiliensis* (Table S1) was extracted from lyophilized leaf tissues using the modified CTAB method as described by Doyle JJ and Doyle JL [27], and the quality and quantity of the obtained DNA were measured by electrophoresis using a 1% agarose gel and spectophotometrically using the NanoDrop ND-1000 (NanoDrop Technologies, Wilmington, DE).

### cDNA library construction and sequencing

Paired-end Illumina mRNA libraries were generated from 4 µg of total RNA following the manufacturer's instructions for mRNA-Seq Sample Preparation (Illumina Inc., San Diego, CA). Library quality was assessed with the 2100 Bioanalyzer (Agilent Technologies, Palo Alto, CA). Cluster amplification was performed using the TruSeq PE Cluster Kit with the cBot automated system (Illumina Inc., San Diego, CA), and each sample was sequenced in separate GAIIx lanes using the TruSeq SBS 36 Cycle Kit (Illumina, San Diego, CA). Read lengths were 72 bp.

### Data filtering and *de novo* assembly

The raw data, which were generated via Illumina sequencing in the BCL format, were converted to qSeq using the Off-Line Basecaller v.1.9.4 (OLB) software. The qSeq files were transformed into FastQ files containing the 72 bp reads using a custom script. The raw reads that were less than 60 bp in length with quality scores of Q<20 were trimmed using the CLC Genomics Workbench (v4.9; CLC Bio, Cambridge, MA). For the *de novo* assembly, we employed the CLC Genomics Workbench with the following parameters: the maximum gap and mismatch count were set to 2, the insertion and deletion costs were set to 3, the minimum contig length was set to 200 bp, the length fraction and similarity parameters were set to 0.5 and 0.9, respectively and the word size (k-mer) was set to 29. All of the short reads were deposited in the NCBI Short Read Archive (SRA) under accession number SRX371361.

### Characterization through similarity searches and annotations

The contigs were searched against the NCBI non-redundant (nr) and the UniProtKB/Swiss-Prot protein databases using BLASTx with a cut-off e-value of 1e-10. The Blast2GO program [28] was used to obtain gene ontology (GO) and Kyoto Encyclopedia of Genes and Genomes (KEGG) annotations. The software WEGO [29] was then employed to perform GO classifications of the annotated contigs to obtain the gene function distributions.

A GO enrichment analysis was conducted to identify the functional categories that were enriched in the bark transcripts. To perform this analysis, we used the Blast2GO program with Fisher's exact test (p-value <0.001).

The contigs were also searched against the STRING database v. 9.05 (http://string-db.org) to predict clusters of orthologous groups (COGs) and classify possible functions at a cut-off e-value of 1e-10. To identify the protein domains, all of the translated sequences were matched against the Pfam database using the InterProScan tool [30].

An *H. brasiliensis* database was constructed using public RNA-seq data [13], [14], [19], [25], the EST database at NCBI (as of August 2013) and data that were provided by Silva et al. (2014) [31] to perform a BLASTn search with a cut-off e-value of 1e-10 for the assessment of the transcriptomic contributions to the publicly available *H. brasiliensis* data and partial and complete open reading frames (ORFs) were predicted using the TransDecoder package (http://transdecoder.sourceforge.net/).

### Digital gene expression analysis

Each genotype was mapped separately to the contigs that were obtained in the *de novo* assembly with a minimum number of reads of 10 and a maximum number of mismatches equal to 2. The data were normalized by calculating the reads per kilobase per million mapped reads (RPKM) for each contig. For the statistical analyses, Kal's Z test on proportions was used to determine the significantly differentially expressed genes. Genes showing false discovery rates (FDR) <0.05 and fold changes >2 were considered to be differentially expressed. All of the analyses were performed with the CLC Genomics Workbench.

### Variant detection

To identify putative SSRs, the MISA program (http://pgrc.ipk-gatersleben.de/misa/) was used. As a criterion for SSR detection, sequences that showed at least 5 dinucleotide repeats; 4 trinucleotide repeats; and 3 tetra-, penta- and hexanucleotide repeats were considered.

The CLC Genomics Workbench software was first used to map the reads to the transcriptome obtained by *de novo* assembly with length fractions of 0.5 and similarities of 0.9. Then, putative SNP detection was performed using the following criteria: minimum coverage of 10, minimum frequency of 10%, quality value from the central base of Q>30 and quality value from the average base of Q>20.

### SNP validation

Primer pairs were designed using the Primer 3 program [32] for at least one putative SNP. PCR amplifications were performed in 20 µl reactions containing 25 ng of genomic DNA, 0.5 µM of each primer, 100 µM of each dNTP, 3 mM MgCl_2_, 20 mM Tris–HCl, 50 mM KCl and 0.5 U of Pfu Taq DNA Polymerase (recombinant) (Thermo Scientific Inc., San Jose, CA) using the following steps: an initial denaturation at 95°C for 3 min, followed by 35 amplification cycles (30 s at 95°C, 30 s at the specific annealing temperature and 2 min at 72°C), and a final extension at 72°C for 10 min. The PCR products were purified using a solution of 20% (w/v) PEG8000 and 2.5 M NaCl in a 1∶1 proportion with the sample volume. The amplification products were resolved via electrophoresis in 1.5% agarose gels prior to the sequencing reaction.

Each amplicon was bidirectionally sequenced (forward and reverse) using the BigDye Terminator v3.1 Kit (Applied Biosystems, Foster City, CA) according to the manufacturer's instructions in an ABI 3500 xL Genetic Analyzer (Applied Biosystems, Foster City, CA). The sequencing chromatograms were visually inspected with the ChromasPro 1.5 software, and SNPs were identified as overlapping nucleotide peaks.

The allelic polymorphic information content of each SNP was calculated using the formula, PIC  =  
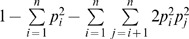
 where *n* is the number of alleles of the marker among the set of genotypes that were used for characterizing the SNP polymorphism, and p*_i_* and p*_j_* are the frequencies of alleles i and j, respectively. The observed and expected heterozygosities were calculated using the TFPGA program [33].

## Results and Discussion

### Transcriptome sequencing and *de novo* assembly

In total, 179,326,804 raw reads were generated and trimmed to exclude low-quality reads (Table 1). To perform the *de novo* assembly 166,731,798 high-quality reads were used, generating 152,416 contigs. The contigs lengths ranged from 97 to 13,266 bp, with a mean length of 536 bp, an N50 of 720 bp and a GC content of 41.8% (Table 2).

**Table 1:** Statistical summary of trimmed Illumina sequencing data.

	n° of reads	average length (bp)	total bases
**Before trimming**			
GT1	85,972,890	68.4	5,880,545,676
PR255	93,353,914	70.2	6,553,444,763
**After trimming**			
GT1	78,512,628	71.6	5,621,504,165
PR255	88,219,170	71.8	6,334,136,406

**Table 2:** Statistical summary of the *de novo* assembly for *H. brasiliensis* bark.

Statistics for the *de novo* assembly	
Contig number	152,416
Total read count	166,731,798
Mean read length	71,76
Mean contig length	536
Maximum contig length	13,266
Minimum contig length	97
N50 length	720
GC% content	41,8

A total of 58,992 contigs longer than 400 bp were selected. Of these, 8,608 shared high identities with non-plant sequences suggesting that 17% of these contigs were contaminant sequences. After removal of these contaminant sequences, a total of 50,384 contigs were used for further analyses (Table S2).

**Figure 1:**
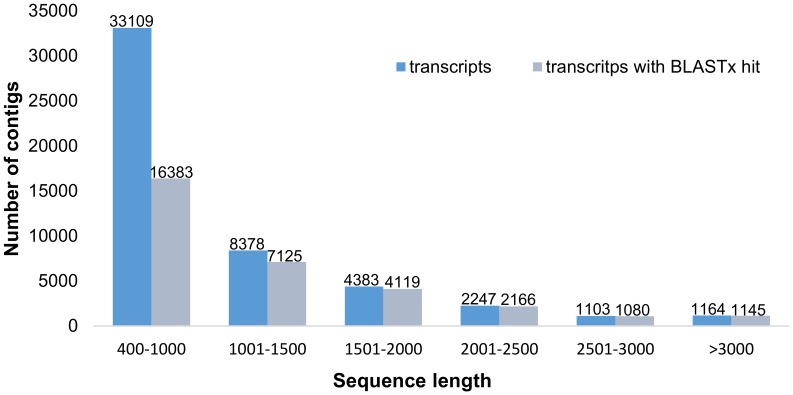
Length distribution of the 50,384 contigs. Histogram of the sequence-length distribution of these transcripts and the transcripts showing BLASTx hits in the nr database with a cut-off e*-*value of 1e−10.

Of the 50,384 contigs, 12,761(25.3%) ranged in size from 1 to 2 kb and 4,515 (8.9%) were longer than 2 kb (Figure 1).

Partial and complete ORFs were predicted from the 50,384 contigs. In total, 23,977 contigs contained ORFs (47.5%), of which 9,247 (18%) were classified as possessing complete ORFs.

### Characterization via similarity searches

The 50,384 contigs were searched against the NCBI nr protein and UniProtKB/Swiss-Prot databases using BLASTx employing a cut-off e-value of 1e-10 as the criterion for defining a significant hit.

Of these contigs, 32,018 (63%) showed significant BLASTx matches in the nr database and 23,620 (47%) in the UniProtKB/Swiss-Prot database (Table 3). All of the contigs that were annotated using UniProtKB/Swiss-Prot were also annotated with the nr database.

**Table 3:** Summary of the annotations of the 50,384 contigs.

Database	Hits	Hits percentage
NCBI non-redundant protein (nr)	32,018	63.54%
UniProtKB/Swiss-Prot	23,620	46.87%
COG	9,720	19.29%
GO	21,725	43.11%
Interpro	16,277	32.30%
KEGG	8,626	17.12%

The proportion of the contigs with BLASTx hits significantly increased for longer contigs (Figure 1). The BLASTx searches yielded hits for 16,383 (49%) contigs that were 400 bp to 1 kb in length, while 4,391 (97%) of the contigs that were longer than 2 kb were annotated in the BLASTx searches. Of the 10 largest contigs, 9 returned BLASTx hits (Table S3).

**Figure 2:**
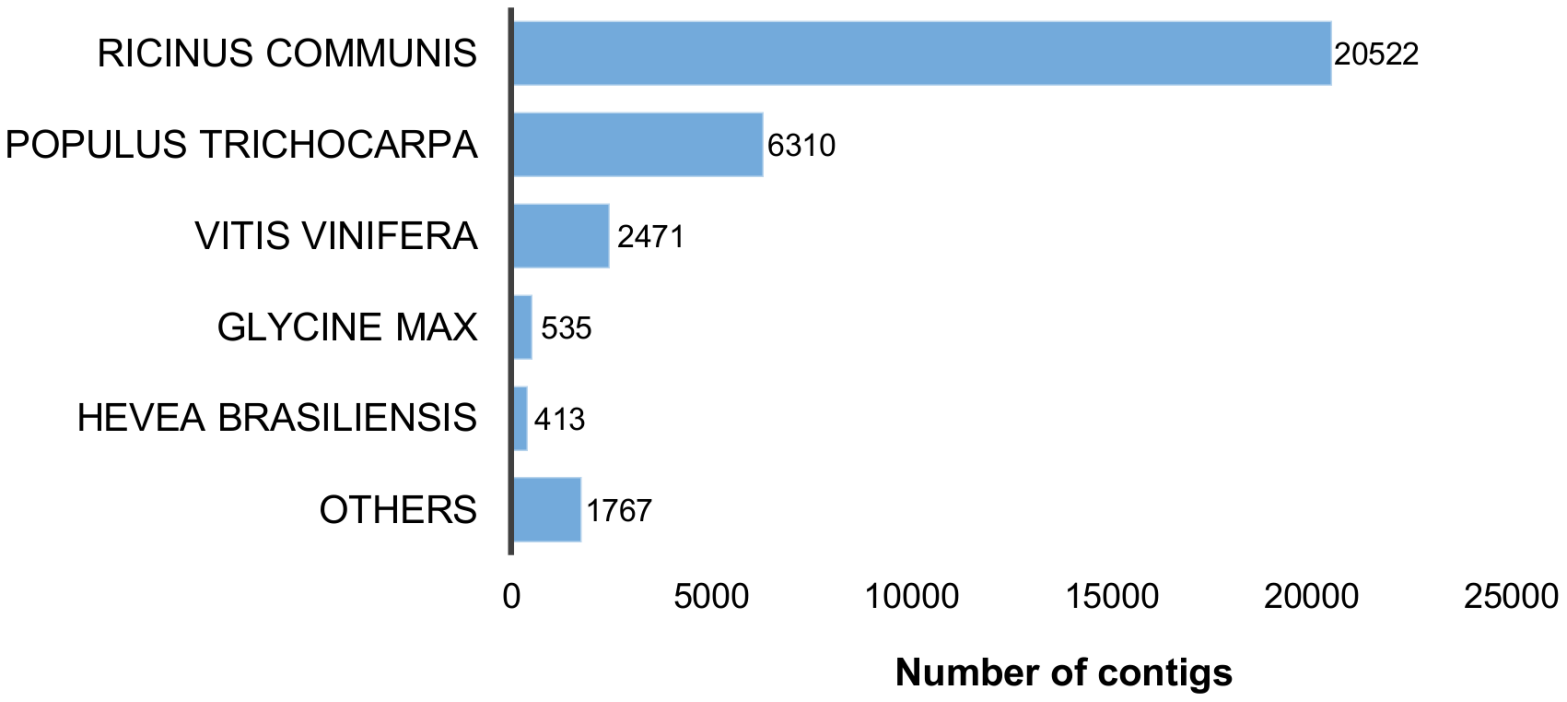
Top-hit species distribution in the BLASTx analysis against the nr database.

The top 5 species showing BLASTx hits were *Ricinus communis* (20,522 contigs; 64%), *Populus trichocarpa* (6,310 contigs; 19.7%), *Vitis vinifera* (2,471 contigs; 7.7%), *Glycine max* (535 contigs; 1.7%) and *Hevea brasiliensis* (414 contigs; 1.3%) (Figure 2).

To investigate the contributions of novel transcripts to the rubber tree database, a BLASTn search (cut-off e-value of 1e-10) was performed against an *H. brasiliensis* database.

Of the 32,018 contigs showing similarity in the nr database, 1,089 (3.4%) non-redundant contigs presented with no hits against the *H. brasiliensis* database (Figure S1). These results indicate that novel uncataloged genes have been identified for the rubber tree database.

Moreover, the 18,866 contigs with no hit that were subjected to BLASTn revealed significant hits for 10,821 (59%), whereas 7,545 (41%) had no hits. A search for putative ORFs was performed with the contigs with no hits (7,545) in BLASTn. We detected 479 contigs with ORFs, of which 83 were classified as complete ORFs (Figure S1). Future analyses may reveal potential unknown genes in this dataset.

### Gene ontology (GO) analysis

The 32,018 contigs showing positive BLAST hits in the nr database were annotated using GO terms. The GO terms allow for the definition and standardization of the properties of gene products in any organism.

**Figure 3:**
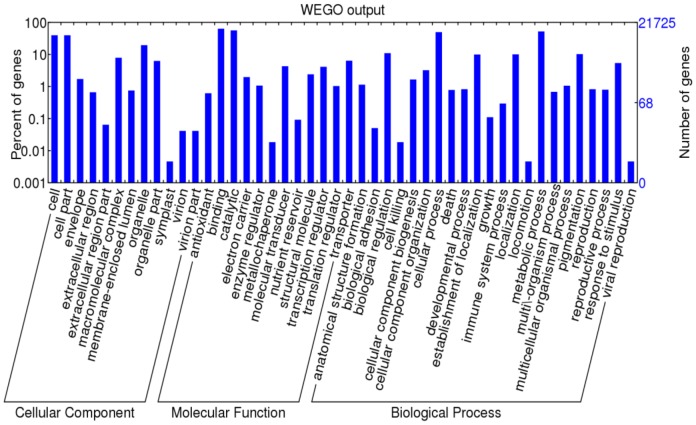
GO classification for the *H. brasiliensis* bark transcriptome.

Of the 32,018 contigs, 21,725 were annotated with 37,781 GO terms (Table 3). Of the three main subontologies, molecular function was the highly represented, with 19,498 contigs followed by biological process with 13,729 contigs and finally, cellular component with 8,686 contigs (Figure 3).

For molecular function, binding (13,547 contigs) and catalytic activity (12,135 contigs) were the highly represented categories. For biological process, metabolic processes (10,528 contigs) and cellular processes (9,953 contigs) figured prominently. Interestingly, 252 contigs were assigned to the category of biological quality regulation, suggesting that they may be related to processes that modulate qualitative or quantitative traits that are associated with biological qualities such as size, mass or shape, which are important characteristics for bark. In addition, 85 contigs were assigned to the category of cell wall organization and thus play roles in the assembly, arrangement of constituent parts or the disassembly of the cell wall. For the cellular component subontology, cells (8,600 contigs) and organelles (4,196 contigs) were the most highly represented.

A GO enrichment analysis was performed to identify the functional categories that were enriched in the bark-exclusive transcripts.

These suggested bark-exclusive transcripts were identified using a BLASTn search (cut-off e-value of 1e-10) against an *H. brasiliensis* database that did not contain bark transcripts.

**Figure 4:**
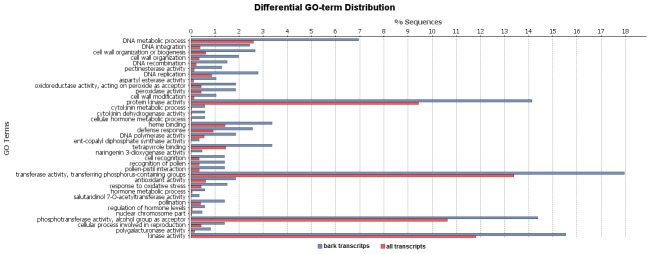
GO enrichment analysis for the bark-exclusive transcripts.

A total of 36 GO terms were enriched (Figure 4) among these transcripts, including the following categories: cell wall organization or biogenesis (GO: 0071554) and cell wall organization (GO: 00771555), which are responsible for the assembly, arrangement of constituent parts or disassembly of cell walls, and cytokinin metabolic (GO: 0009690) processes which are related to plant growth.

Categories that are involved in the prevention and/or recovery from an infection that is caused by an attack, such as the defense response (GO: 0006952) and pectinesterase activity (GO: 0030599) were also enriched.

### Clusters of orthologous groups (COGs)

**Figure 5:**
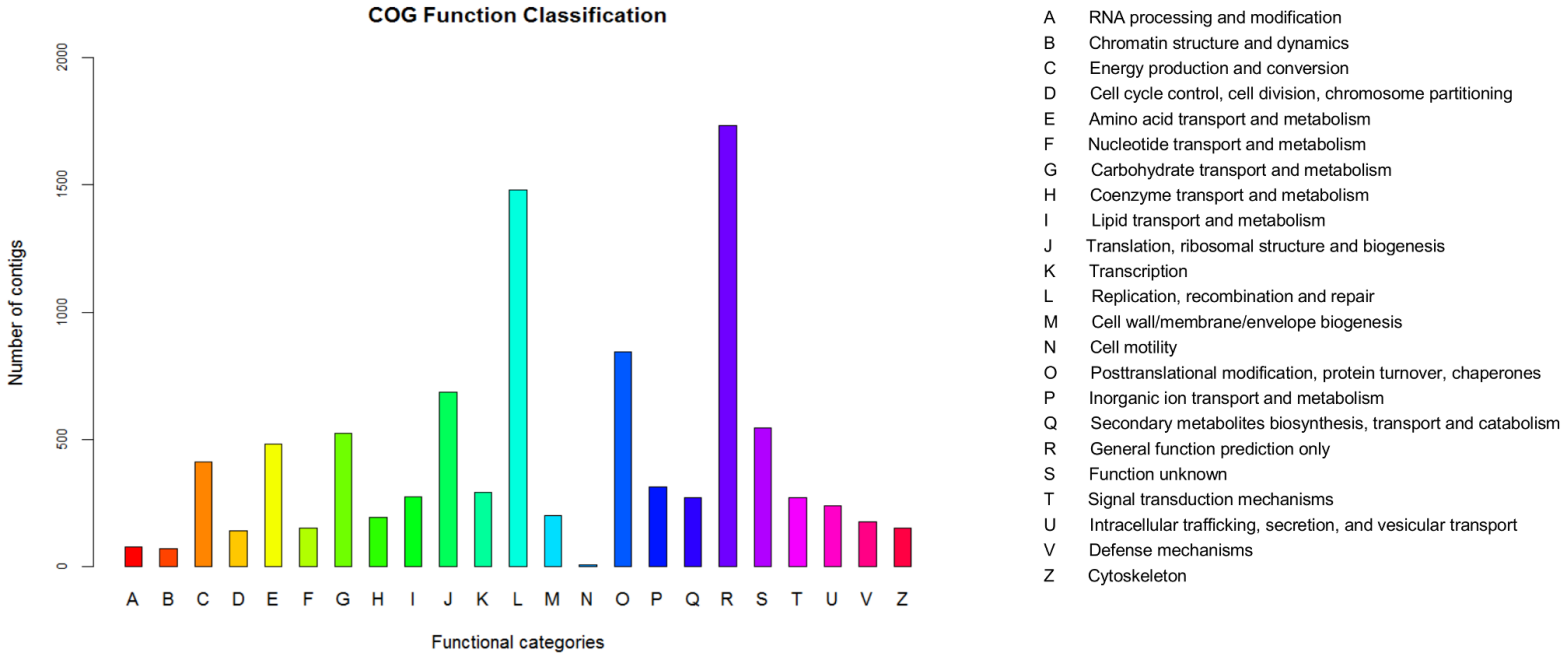
COG functional distribution of the *H. brasiliensis* bark transcriptome.

The clusters of orthologous groups (COGs) of protein database is used to phylogenetically classify the proteins that are encoded in complete genomes. Each COG includes proteins that are inferred to be orthologs i.e., they are direct evolutionary counterparts [34]. Among the 50,384 contigs, 9,720 were annotated (Table 3) and classified into 23 COG categories (Figure 5). General function prediction was the most highly represented category with 1,732 contigs, followed by replication, recombination and repair with 1,480 contigs and posttranslational modification, protein turnover, and chaperones with 843 contigs.

The smallest groups that were observed in the COG annotation analysis were cell motility, chromatin structure and dynamics and RNA processing and modification (7, 69 and 77 annotated contigs, respectively).

Additionally, the category of secondary metabolite biosynthesis, transport and catabolism was represented by 270 contigs.

### Protein domain analysis

A comparison of the 50,384 contigs against the Pfam domain database with a cut-off e-value of 1e-10 resulted in 16,277 contigs matching at least one protein domain model (Table 3). The distribution of the domains ranged from a minimum of one to a maximum of 34 domains per contig.

**Figure 6:**
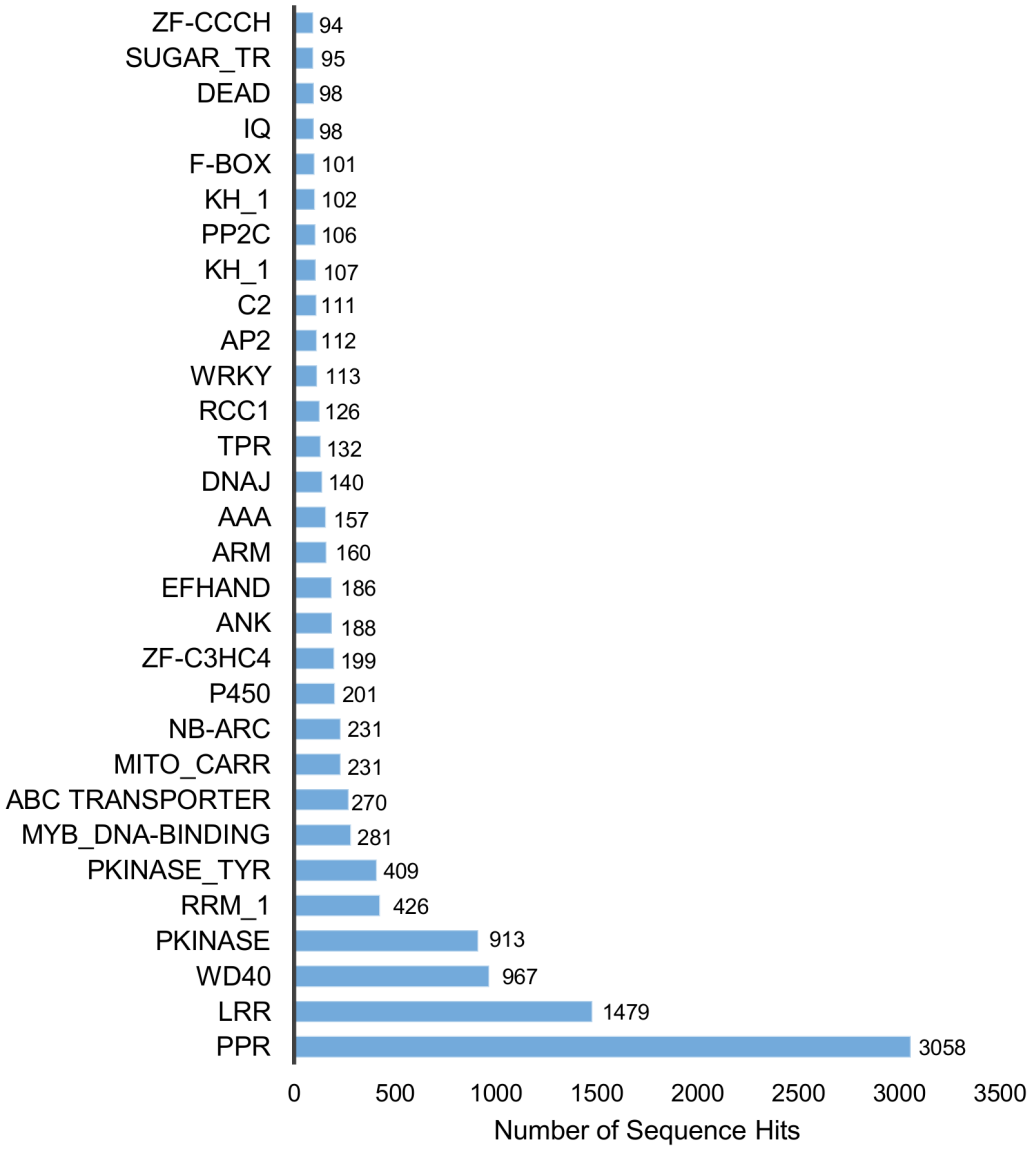
Distribution of the top 30 Pfam domains identified in translated *H. brasiliensis* transcripts.

The 3 most abundant domains that were identified included pentatricopeptide repeat-containing proteins (PPRs) with 3,058 contigs, followed by leucine-rich repeats (LRRs) with 1,479 contigs and WD40 with 967 contigs. The WD40 domain functions as a site of protein-protein interaction, and proteins containing WD40 repeats are known to serve as platforms for the assembly of protein complexes or mediators of transient interplay among other proteins [35] (Figure 6). Furthermore, 112 contigs were associated with WRKY domains which is a DNA-binding transcription factors tnat are found almost exclusively in plants [36] (Figure 6). WRKY containing proteins are thought to play important roles in plant defense responses, plant hormone signaling, secondary metabolism and plant responses to abiotic stress [37].

Moreover, 95 contigs were annotated to the sugar transporter family (Figure 6), 49 to the cellulase synthase family and 11 to cellulase domains (data not shown).

### KEGG classification

The KEGG pathways represent collections of manually drawn pathway maps and that are helpful for the understanding if the biological functions and interactions of genes [38], [39].

Of the 21,725 contigs that were annotated with GO terms, 8,626 were assigned to 10,355 EC numbers (Table 3). These EC numbers were mapped to the 137 KEGG Pathways (Table S4). Of the 5 main categories, metabolism was the main category represented, with 92% followed by organismal systems, environmental information and genetic information processing with 5%, 2% and 1% respectively.

**Figure 7:**
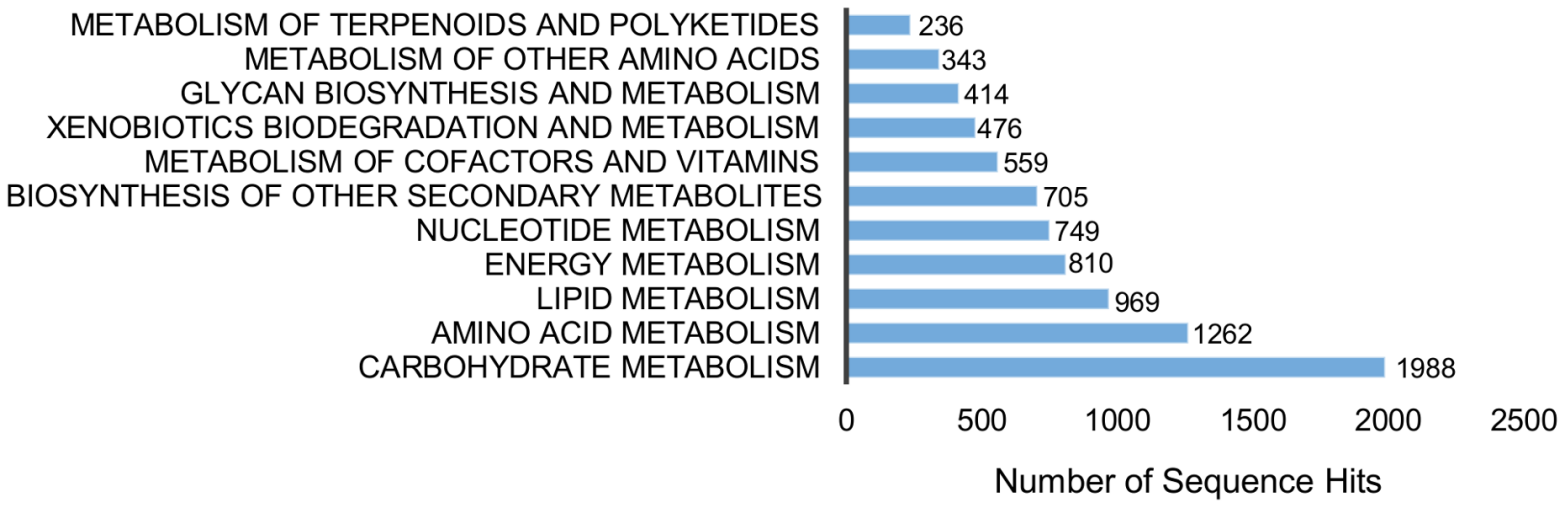
KEGG metabolism pathway distribution for the *H. brasiliensis* contigs.

In the metabolism category, carbohydrate metabolism (1,988 contigs) and amino acid metabolism (1,262 contigs) were the most prominent classes (Figure 7).

#### Rubber biosynthesis pathway

Latex is produced in specialized cells known as laticifers or latex vessels, which are located adjacent to the phloem of the rubber tree [4]. The chemical composition of rubber includes high-molecular-weight cis-polyisoprene [1], which is formed through the sequential condensation of isopentenyl diphosphate (IPP) [17]. IPP biosynthesis is related to the mevalonate (MVA) pathway [4], which occurs in the cytoplasm, and the 2-C-methyl-D-erythritol 4-phosphate (MEP) pathway which occurs in the plastid [18].

The MVA pathway includes 6 steps, which are catalyzed by the 6 corresponding enzymes, whereas the MEP pathway is catalyzed by 7 enzymes [4], [18]. IPP that is synthetized through the MEP pathway was initially thought to be used for carotenoid synthesis in Frey-Wyssling particles [40]. However, the MEP pathway has been shown to serve as an alternative source of IPP for cis-polyisoprene synthesis in mature rubber trees or in clones that do not produce a large amount of carotenoids [18].

Acetyl-CoA is a precursor of the MVA pathways and is produced through the glycolysis/gluconeogenesis pathway. The MEP pathway precursors include glyceraldehyde-3-phosphate, which is produced via the glycolysis/gluconeogenesis pathway, and pyruvate, which is a product of pyruvate metabolism.

For the KEGG annotations, 192 contigs were annotated to 25 enzymes in the glycolysis/gluconeogenesis pathway (Figure S2), and 116 were annotated to 22 enzymes in pyruvate metabolism (Figure S3).

In addition, we identified all of the key genes that are involved in the MVA and MEP pathways through KEGG annotations (Figure S4). In total, 25 contigs were related to the MVA pathway, and 40 were related to the MEP pathway (Table 4).

**Table 4:** Number of contigs annotated in the MVA and MEP pathways.

MVA pathway	
Enzymes	number of contigs
acetyl-CoA C-acetyltransferase (AACT)	4
hydroxymethylglutaryl-CoA synthase (HMGS)	4
hydroxymethylglutaryl-CoA reductase (NADPH)	8
mevalonate kinase (MVK)	3
phosphomevalonate kinase (PMK)	2
diphosphomevalonate decarboxylase (MVD)	4

MEP pathway	
Enzymes	number of contigs
1-deoxy-D-xylulose-5-phosphate synthase (DXS)	18
1-deoxy-D-xylulose-5-phosphate reductoisomerase (DXR)	3
2-C-methyl-D-erythritol 4-phosphate cytidylyltransferase (MCT)	2
4-(cytidine 5′-diphospho)-2-C-methyl-D-erythritol kinase (CMK)	3
2-C-methyl-D-erythritol 2,4-cyclodiphosphate synthase (MDS)	2
(E)-4-hydroxy-3-methylbut-2-enyl-diphosphate synthase (HDS)	5
4-hydroxy-3-methylbut-2-enyl diphosphate reductase (HDR)	4

### Digital gene expression analysis

We conducted a gene expression analysis to evaluate the potential genes that were differentially expressed between the GT1 and PR255 genotypes.

In this analysis, we observed that 716 genes were expressed at higher levels in GT1, and 1,267 were more prominently expressed in PR255 (Figure S5)

The top 20 differentially expressed genes that were found for each genotype are listed in Table S5. Similar to Li et al. (2012) [14], we observed genes that were related to stress/defense responses, such as the chalcone synthase [41], glycine-rich RNA-binding protein [42], ascorbate peroxidase [43] and o-methyltransferase [44] genes, as these clones were frequently harvested.

Interestingly, the gene enconding carbonic anhydrase was the most highly expressed in PR255. This enzyme is responsible for facilitating the diffusion of carbon dioxide in photosynthesis and is essential for processes such as respiration [45].

The most highly expressed gene in GT1 was phenylalanine ammonia-lyase 2 which is involved in lignin and flavonoid synthesis and is typically highly expressed in response to pathogen attack and tissue wounding [46].

Considering the annotations of all the key genes that are involved in the MVA and MEP pathways according to the KEGG database, we observed that the genes encoding hydroxymethylglutaryl-CoA reductase (NADPH) (contig_104848) in the MVA pathway and (E)-4-hydroxy-3-methylbut-2-enyl-diphosphate synthase (contig_145940 and contig_146058) and 4-hydroxy-3-methylbut-2-enyl diphosphate reductase (contig_97647) in the MEP pathway, were enhanced in the GT1 genotype, providing a strong evidence that these genes are differentially expressed in these two clones.

Although the experiments did not include replicates, this analysis represents the first step in understanding the unique responses of different genotypes and elucidating possible candidate genes for rubber tree molecular breeding.

### Putative SSR marker discovery

The 50,384 contigs were subjected to a search for putative SSR markers. A total of 17,927 SSRs were detected in 13,070 contigs, and 3,433 contigs presented with more than one SSR (Table 5). There were 6,822 di-, 6,098 tri-, 3,033 tetra-, 1,125 penta- and 849 hexanucleotide potential SSRs (Table 6). A total of 50,608,451 bp were analyzed, and a frequency of one SSR per 2.8 kb was observed, similar to previously described by Feng et al. (one SSR per 2.25 kb) [12] and by Li et al. (one SSR per 2.42 kb) [14].

**Table 5:** Summary of putative SSRs identified using MISA software.

Number of contigs	50,384
Total bases	50,608,451
Number of sequences with SSRs	13,070
Total number of SSRs	17,927
SSR frequency	1 per 2.8 kb

**Table 6:** Summary of the distribution of putative SSR motifs.

SSR repeats	3	4	5	6	7	8	9	10	11	12	13	14	15	>15	Total
AC/GT	-	-	364	113	72	56	54	35	34	27	19	13	12	30	829
AG/CT	-	-	1,702	677	362	272	277	307	341	174	109	107	102	244	4,674
AT/AT	-	-	549	240	149	111	66	61	38	46	13	11	7	7	1,298
CG/CG	-	-	9	7	3	2	-	-	-	-	-	-	-	-	21
dinucleotide	-	-	2624	1037	586	441	397	403	413	247	141	131	121	281	6,822
AAC/GTT	-	242	54	19	9	6	5	1	1	-	-	-	-	3	340
AAG/CTT	-	978	369	202	104	99	33	41	29	9	5	5	2	-	1,876
AAT/ATT	-	390	174	104	80	64	22	19	17	10	10	6	4	5	905
ACC/GGT	-	363	108	54	25	7	8	3	2	-	-	-	-	-	570
ACG/CGT	-	61	14	5	2	1	2	-	1	-	-	-	-	-	86
ACT/AGT	-	44	15	2	1	1	-	1	-	-	-	-	-	1	65
AGC/CTG	-	445	131	68	23	13	2	3	-	-	-	-	-	-	685
AGG/CCT	-	360	108	53	27	21	3	2	2	-	-	-	-	-	576
ATC/ATG	-	607	175	57	34	17	7	7	1	-	-	-	-	-	905
CCG/CGG	-	56	22	10	2	-	-	-	-	-	-	-	-	-	90
trinucleotide	-	3,546	1,170	574	307	229	82	77	53	19	15	11	6	9	6,098
tetranucleotide	2456	385	142	34	13	2	1	-	-	-	-	-	-	-	3,033
pentanucleotide	860	205	44	6	6	3	1	-	-	-	-	-	-	-	1,125
hexanucleotide	625	157	49	16	1	1	-	-	-	-	-	-	-	-	849

To investigate the contributions of the novel sequences containing SSRs for future rubber tree studies, we performed a BLASTn search using our identified sequences with SSRs against the *H. brasiliensis* database. We identified 1,709 sequences that showed no similarity with the *H. brasiliensis* database, suggesting that they possess novel SSRs for the rubber tree, and thus 203 sequences were annotated with the nr database.

Dinucleotide SSRs have been reported to be the most abundant SSR type in plant genomes [47]. In contrast with plants such as the sugarcane [48], wheat [49], sweet potato [50] and citrus [51], in which SSRs containing trinucleotide motifs are the most abundant in transcribed regions, it has been reported that dinucleotide motifs figure prominently in *H. brasiliensis* transcripts [12]. Dinucleotide motifs are also abundant in other plants such as sesame [52], kiwi [53] and coffee [54]. In this work, dinucleotide motifs were found to be predominant, corroborating with previous studies in which 38% of the total putative SSRs were shown to possess these motifs (Figure S6).

The most abundant motif in the dinucleotide class was AG/TC (4.674, 68.5%), followed by AT/TA (1298, 19%), AC/TG (829, 12.1%) and GC/CG (21, 0.3%) (Figure S6). The rarity of the CG dinucleotide microsatellites cannot be explained by the low C/G contents. CpG dinucleotides that are not situated in CpG islands can undergo cytosine methylation, and methylated cytosines tend to mutate to thymine, which may explain the underrepresentation of the CpG dinucleotides and, consequently, the low coverage of microsatellites CG motifs [55]. The most frequent trinucleotide motif was AAG/TTC (1876, 30.7%), and the least represented motif was CCG/CGG (90, 1.4%) (Table 6). Previous studies on *Arabidopsis* and soybean also suggested that the trinucleotide AAG motif may figure prominently in dicots [50]. Interestingly, we found only 90 CCG/CGG trinucleotides, which have been reported to predominant in monocots [47], [56], such as maize, barley and sorghum [50]. Our results are in accord with previous studies if rubber tree and with the observed rarity of CCG/CGG repeat units that have been reported in a large number of dicotyledonous plants such as *Citrus*, *Coffea* and *Glycine* [57]. Long CCG/CGG sequences could compete with the components of the splicing machinery, resulting in inadequate splicing. Moreover, CCG/CGG repeats, may potentially form higher structures, such as hairpins and quadruplexes, affecting the efficiency and accuracy of splicing and influencing the formation of mature mRNA [56], [58].

Our findings correlate with previous studies of the rubber tree, in which AG/TC and AAG/TTC were found to be the most abundant motifs in the dinucleotide and trinucleotide categories, respectively.

### Putative SNP marker discovery

For the putative SNP detection, the 50,384 contigs were first mapped with trimmed short sequence reads using the CLC Genomics Workbench. In total, 404,114 putative SNPs were detected, and an average of one SNP per 125 bp was observed (Table 7), which was similar to the SNP frequencies that were previously reported for *Eucalyptus grandis* (1 SNP per 192 bp) [59], apple (1 SNP per 149 bp) [60] and grapevine (1SNP per 117 bp) [61], in addition to a recent study for rubber tree (1 SNP per 178 bp) [31]. However, the density of putative SNPs was higher than that which was described by Pootakham et al. (2011) [62] and Salgado et al [63] for the rubber tree, who detected one SNP per 1.5 kb and one SNP per 5.2 kb, respectively, using 454 pyrosequencing technology, which has a lower sequencing depth than Illumina sequencing technology.

**Table 7:** Summary of putative SNPs identified using CLC Genomics Workbench.

Number of contigs	50,384
Total bases	50,608,451
Number of SNPs	404,114
SNP frequency	1 per 125 bp
Transition	242,732
A ↔ G	120,866
C ↔ T	121,866
Transversion	161,382
A ↔ C	40,681
A ↔ T	49,289
C ↔ G	31,376
G ↔ T	40,036

Transition SNPs were predominant, of which 242,732 (60%) were detected, while 161,382 (40%) transversion SNPs were identified (Table 7). Among the transversion variations, A ↔ T was the most highly represented with 49,283 SNPs detected, and G ↔ C was the least common with 31,376 SNPs identified (Figure S7).

As expected, the transition SNPs were generally observed at higher frequencies than the transversion SNPs. During natural selection, transitions mutations are better tolerated than transversions because they generate synonymous mutations in protein-coding sequences [64].

Because contigs corresponding with genes that are involved in the MVA and MEP pathways were identified in the KEGG annotations, we also searched for SNPs in these sequences. Only 4 contigs that are involved in the MVA pathway did not contain putative SNPs, which were annotated as hydroxymethylglutaryl-CoA synthase (AACT) (1 contig), hydroxymethylglutaryl-CoA reductase (NADPH) (2 contigs) and phosphomevalonate kinase (PMK) (1 contig), while 1 contig from the MEP pathway that did not contain a putative SNP was found, which was annotated as 1-deoxy-D-xylulose-5-phosphate synthase (DXS).

### SNP marker validation

Primer pairs were designed for the sequences that were related to the MVA and MEP pathways with putative SNPs to validate the SNPs via Sanger sequencing.

We designed primer pairs for 21 and 31 transcripts from the MVA and MEP pathways, respectively. For some of the sequences, we designed more than one primer pair to validate a greater number of SNPs.

A total of 64 primer pairs were designed and 35 yielded good amplification products for sequencing. However, 9 loci yielded good amplification products in only a few genotypes, and 26 loci were therefore analyzed for SNP marker validations. Some of the loci showed deviations from the expected and observed product sizes because the primers pairs were designed based on transcript regions (exons), whereas the amplification reactions were performed with genomic DNA which contains both exons and intron regions (Table S6).

A total of 78 SNPs were validated in 25 contigs (Table S7). Of these 25 contigs, 9 were annotated to the MVA pathway. Among the 6 enzymes in the MVA pathway, we amplified transcripts that were annotated as the enzymes acetyl-CoA C-acetyltransferase (AACT) (1 contig; 2 SNPs), hydroxymethylglutaryl-CoA synthase (HMGS) (2 contigs; 2 SNPs), hydroxymethylglutaryl-CoA reductase (NADPH) (5 contigs; 12 SNPs) and diphosphomevalonate decarboxylase (MVD) (1 contig; 1 SNP). For the MEP pathway, we evaluated 14 contigs that were annotated as the enzymes 1-deoxy-D-xylulose-5-phosphate synthase (DXS) (10 contigs; 53 SNPs), 1-deoxy-D-xylulose-5-phosphate reductoisomerase (DXR) (1 contig; 1 SNP), 2-C-methyl-D-erythritol 2,4-cyclodiphosphate synthase (MDS) (2 contigs; 6 SNPs) and (E)-4-hydroxy-3-methylbut-2-enyl-diphosphate synthase (HDS) (1 contig; 1 SNP). The observed and expected heterozygosities ranged from 0.0294 to 0.9167 and from 0.0000 to 0.5394 respectively, and the PIC values ranged from 0.0286 and 0.4402.

**Figure 8:**
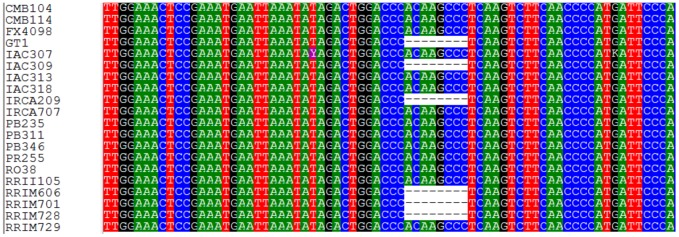
INDEL polymorphism at the HB_SNP_26 locus.

Interestingly, the locus HB_SNP_26 which was annotated as diphosphomevalonate decarboxylase (in the MVA pathway), contained a deletion or insertion (INDEL) polymorphism from positions 161 to 168 bp (Figure 8).

The observed and expected heterozygosities and PIC values were not calculated to the INDEL polymorphisms.

This study provides the first identification and validation of putative SNPs in 2 important pathways for rubber biosynthesis.

### RNA-seq for *H. brasilensis* breeding

Crop domestication began more than 10,000 years ago, but the domestication and breeding of forest trees, such as rubber tree, only started approximately 100 years ago. Similar to other forest tree species with long generation times, rubber tree still in the early stages of domestication, with most breeding programs producing only two or three generations from the wild-type genotypes [5], whereas the same amount of progress can be accomplished in a single year for many agricultural crops [65].

With the advent of next-generation sequencing technologies, such as RNA-seq, rapid advances have been made in improving the levels of transcriptome coverage for forest trees. These transcripts can be characterized using public databases, and an enormous amount of genetic diversity has been identified in these species.

Since 2011, the publically available RNA-seq data [13], [14], [19], [25] have included an abundance of new information provided on *H. brasiliensis* transcripts and, consequently on rubber tree genetics [25]. These data allowed us to compare and identify novel transcripts (Figure S1) and new sequences with SSRs for the *H. brasiliensis* database to improve this database.

The high genetic variability that is present in *H. brasiliensis* have been demonstrated by the high frequency of polymorphisms that are found in its SSR [11], [66], [67] and EST-SSR [12], [31] markers. SNP markers constitute the most abundant type of DNA polymorphism in genomic sequences and are thought to play major roles in the induction of phenotypic variations [68]. RNA-seq, together with SNP discovery, can be applied to develop new markers in candidate genes for genetic breeding and to investigate the variability of these genes in rubber tree, which has been performed in other tree species. The integration of modern genetics and novel sequencing technologies with conventional breeding can provide additional information and should expedite *H. brasiliensis* domestication.

## Conclusions

The use of RNA-seq technology has allowed for a more comprehensive understanding of transcriptional patterns occurring in the bark of *H. brasiliensis*. Furthermore, our data has revealed 1,089 new rubber tree genes and 7,545 potentially novel genes. The RNA-seq data has led to the identification of 1,709 new EST-SSRs for the *H. brasiliensis* database. In addition, SNP analysis elucidated a total 404,114 SNPs that may be associated with potentially important genes. This information may constitute a valuable resource for rubber tree breeding programs and genetic diversity studies. This is the first study in which putative SNPs were identified and validated in genes that are involved in the MVA and MEP pathways.

## Supporting Information

Figure S1 **Overview of the workflow for investigating the contribution of novel transcripts in the *H. brasiliensis* database.**
(TIFF)

Figure S2 **Glycolysis/gluconeogenesis KEGG pathway.** The annotated contigs are indicated in yellow.
(TIFF)

Figure S3 **Pyruvate metabolism KEGG pathway.** The annotated contigs are indicated in yellow.
(TIFF)

Figure S4 **MVA and MEP KEGG pathways.** The annotated contigs are indicated in yellow.
(TIFF)

Figure S5 **Digital gene expression analysis.** Volcano plot of differentially expressed genes between the GT1 and PR255 genotypes.
(TIFF)

Figure S6 **Distribution of putative microsatellite types.**
(TIF)

Figure S7 **Distribution of putative SNPs that were identified.**
(TIF)

Table S1 **Genotypes of** ***H. brasiliensis*** **that were used for SNP validations and characterizations.**
(XLSX)

Table S2 **The 50,384 contigs that were longer than 400 bp from the** ***de novo*** **assembly.**
(XLSX)

Table S3 **The 10 longest contigs from the** ***de novo*** **assembly.**
(XLSX)

Table S4 **The 137 pathways that were annotated in the KEGG database.**
(XLSX)

Table S5 **The 20 most highly expressed genes in the GT1 and PR255 genotypes.**
(XLSX)

Table S6 **Characterization of all the developed SNP markers.** The table presents the SNP markers that were developed for *H. brasiliensis*, including the corresponding primer sequences, annealing temperatures, and expected and observed products sizes in 1.5% agarose gel electrophoresis.
(XLSX)

Table S7 **Validation of the SNP markers.** The table presents the allelic variants, observed and expected heterozygosities and polymorphism information contents.
(XLSX)
