# Supplementary figures and images for: De Novo Assembly and Transcriptome Analysis of the Rubber Tree (Hevea brasiliensis) and SNP Markers Development for Rubber Biosynthesis Pathways
Source: PLoS One. 2014 Jul 21;9(7):e102665. doi: 10.1371/journal.pone.0102665 (PMC4105465; doi:10.1371/journal.pone.0102665)

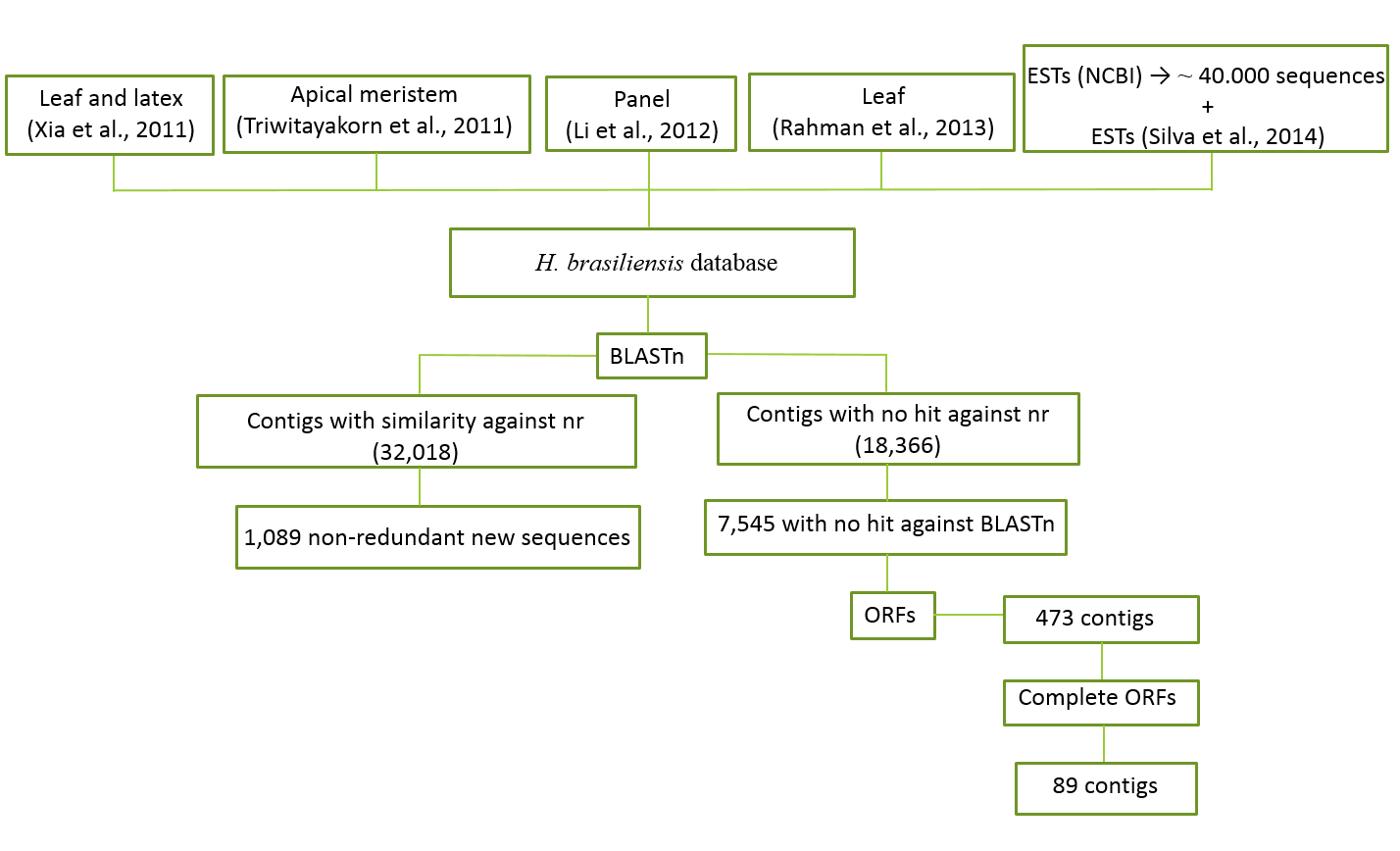

Supplement: Figure S1 — Overview of the workflow for investigating the contribution of novel transcripts in the H. brasiliensis database. (TIFF) [file pone.0102665.s001.tiff]

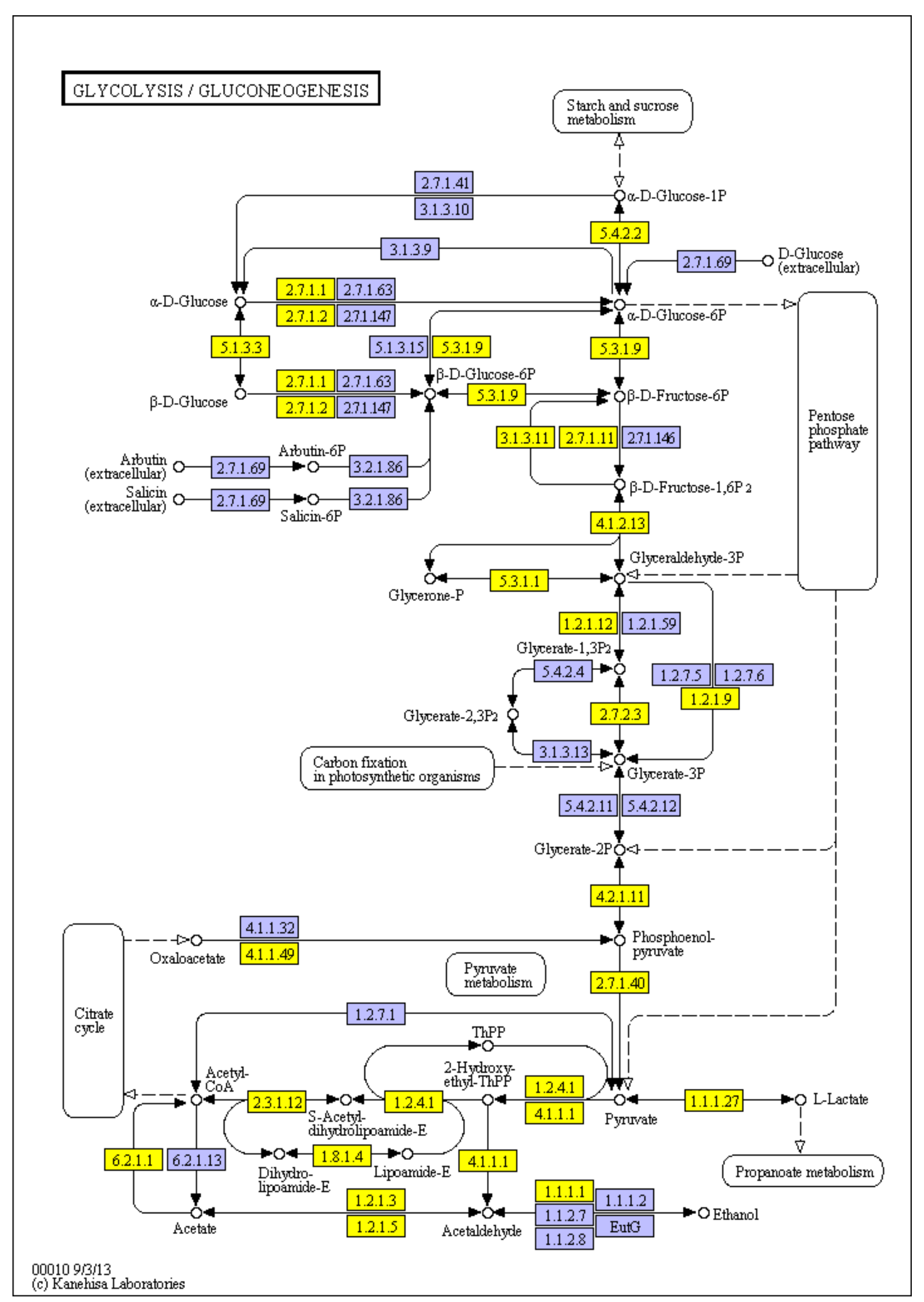

Supplement: Figure S2 — Glycolysis/gluconeogenesis KEGG pathway. The annotated contigs are indicated in yellow. (TIFF) [file pone.0102665.s002.tiff]

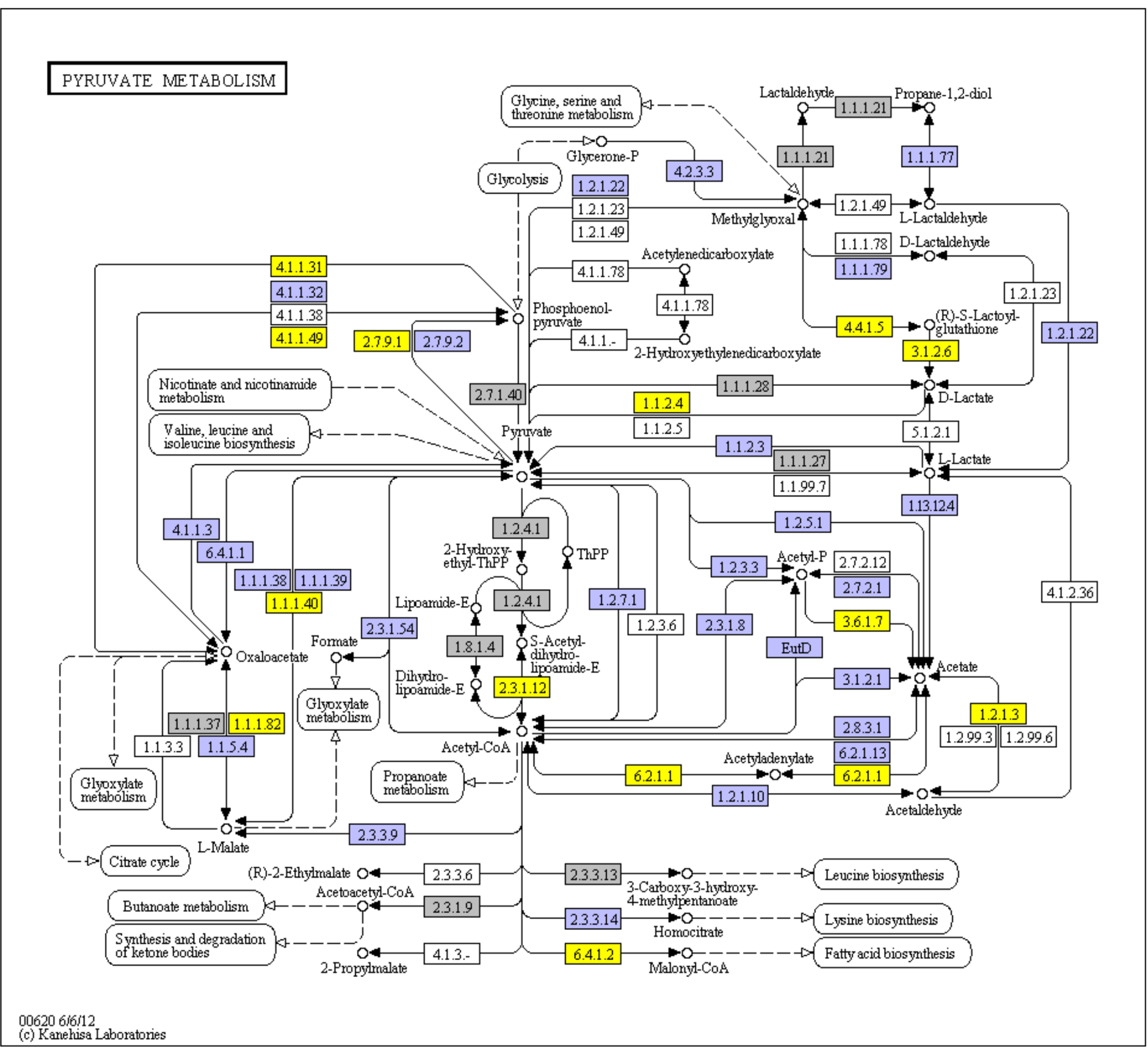

Supplement: Figure S3 — Pyruvate metabolism KEGG pathway. The annotated contigs are indicated in yellow. (TIFF) [file pone.0102665.s003.tiff]

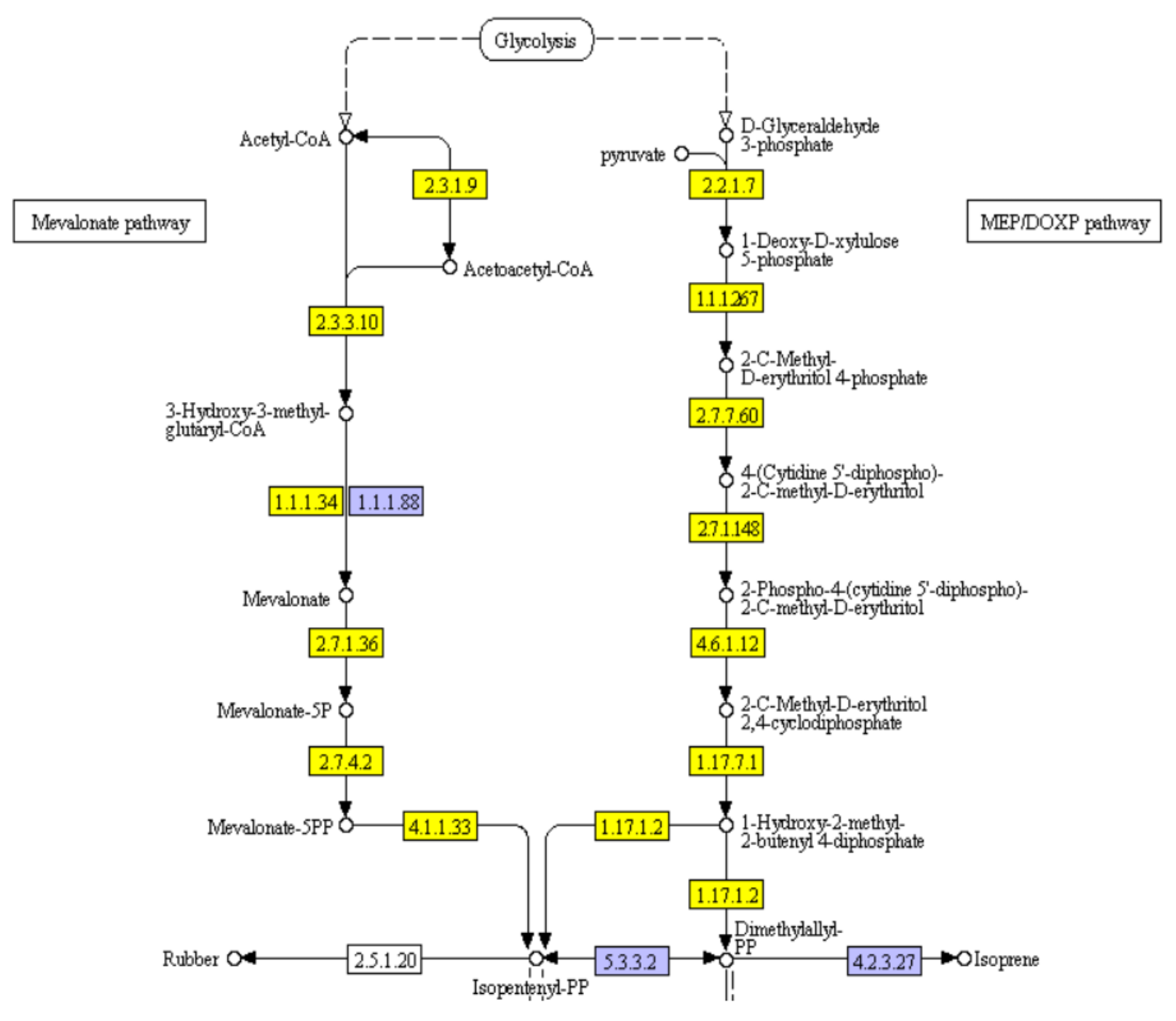

Supplement: Figure S4 — MVA and MEP KEGG pathways. The annotated contigs are indicated in yellow. (TIFF) [file pone.0102665.s004.tiff]

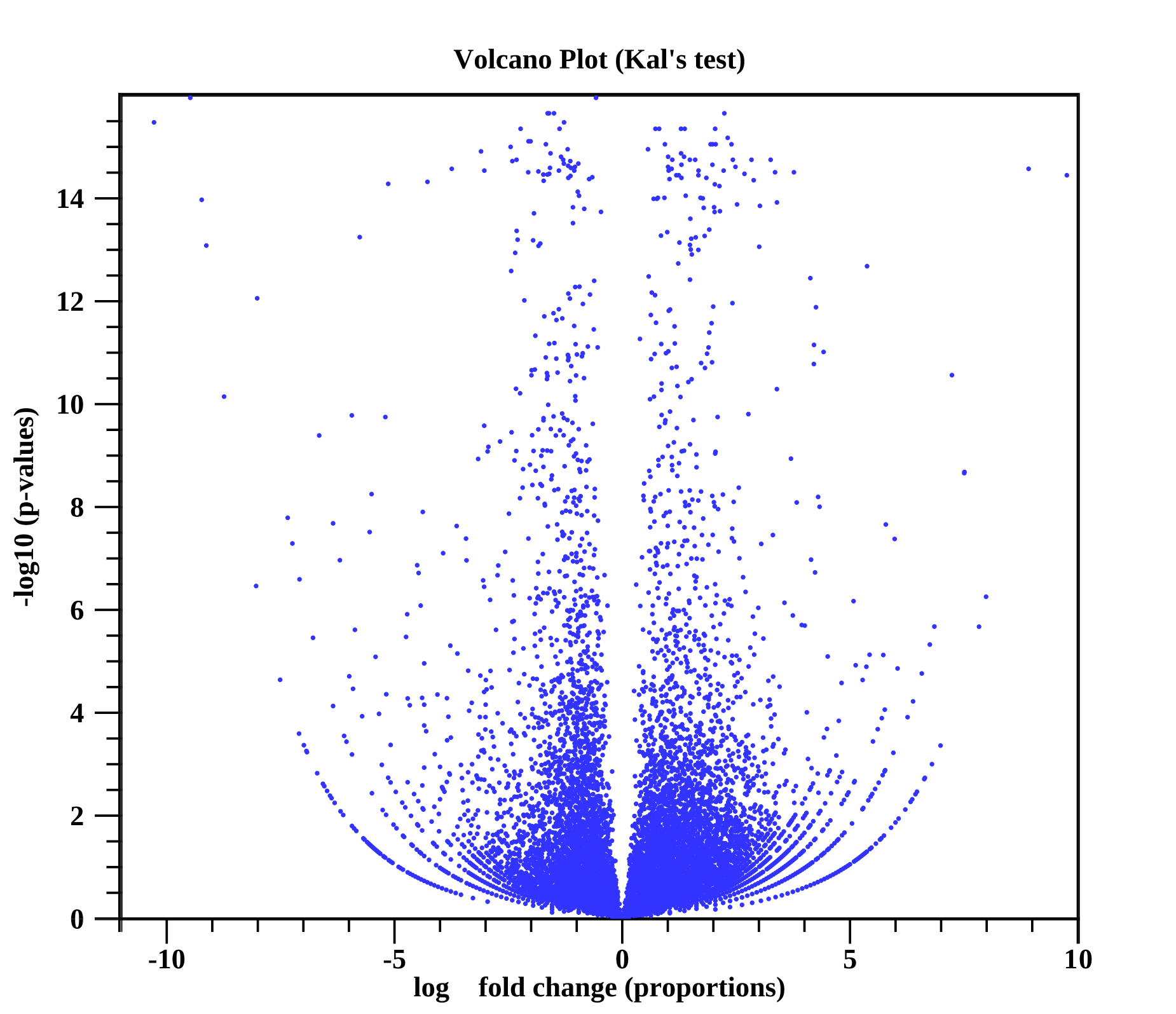

Supplement: Figure S5 — Digital gene expression analysis. Volcano plot of differentially expressed genes between the GT1 and PR255 genotypes. (TIFF) [file pone.0102665.s005.tiff]

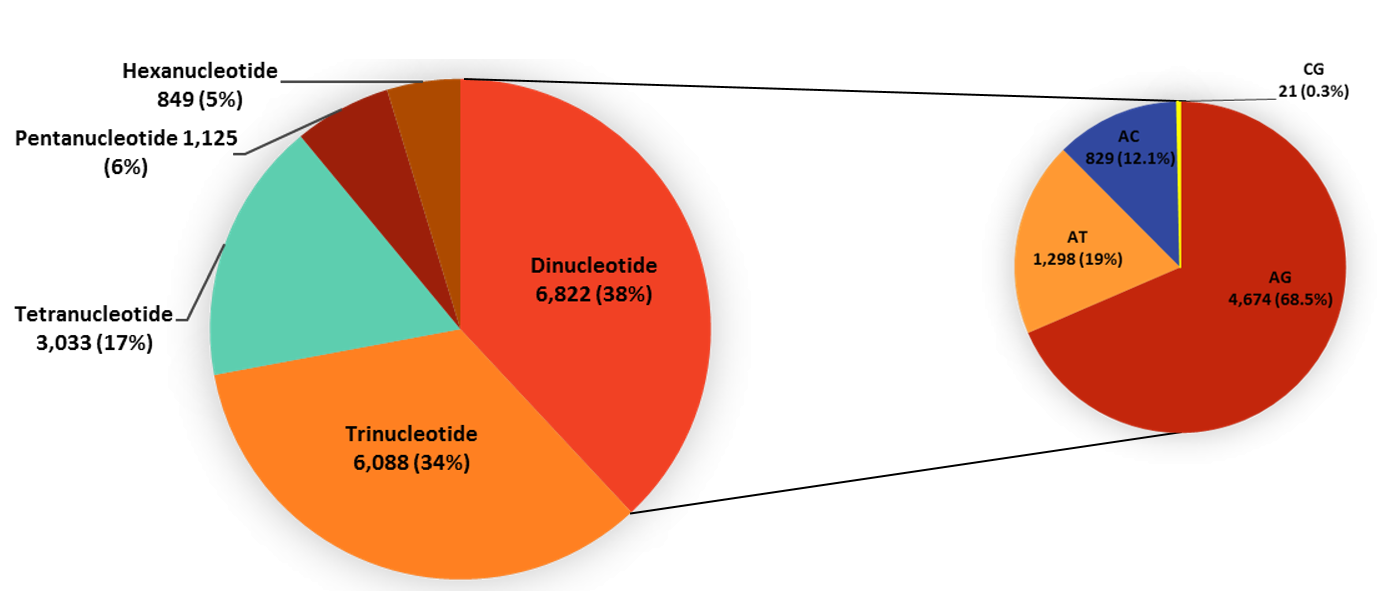

Supplement: Figure S6 — Distribution of putative microsatellite types. (TIF) [file pone.0102665.s006.tif]

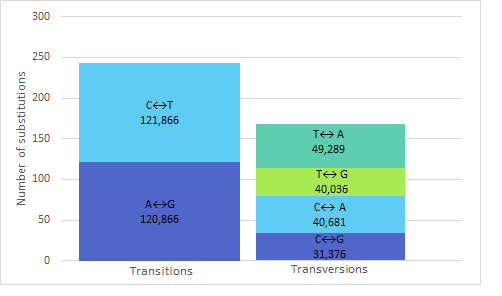

Supplement: Figure S7 — Distribution of putative SNPs that were identified. (TIF) [file pone.0102665.s007.tif]
